# Alan Fincham and the era of enamel protein Biochemistry

**DOI:** 10.3389/fphys.2022.1071265

**Published:** 2022-12-05

**Authors:** Thomas G. H. Diekwisch

**Affiliations:** Department of Oral and Craniofacial Sciences Margaret and Cy Welcher Professor of Dentistry, Eastman Institute of Oral Health, University of Rochester School of Medicine and Dentistry, Rochester, NY, United States

**Keywords:** amelogenin, TRAP, LRAP, nanospheres, problem-based learning

## Abstract

Enamel research experienced an unprecedented period of growth during the latter part of the 20th century until today. This growth is in part due to the contributions of a number of iconic scientists such as Alan G. Fincham, the focus of the present review. Alan was involved in many of the seminal discoveries of this time, including the identification of the critical amelogenin peptides TRAP and LRAP, the determination of the amelogenin amino acid sequence, the identification of the sole serin-16 phosphorylation site, and the amelogenin nanosphere theory. Alan was also a superb mentor to graduate students and others. His experience and leadership related to problem-based learning greatly affected predoctoral dental education at the University of Southern California and in the United States.

## Introduction

During its early decades, enamel research has been an unusual discipline, with individual researchers scattered across the globe and international meetings only held every five to 10 years. These “enamel meetings” have been numbered so far from Enamel I to Enamel X, and scientific findings have been printed in proceedings, with the paper discussions recorded and transcribed in painstaking detail. Many scientists in the early years of enamel research thought that those transcribed discussions exceeded the value of the printed scientific papers.

During the mid-20th century, enamel research focused on the impact of caries on tooth enamel loss and on the unusually symmetric morphology of enamel crystals. Common techniques used to study enamel morphology included scanning electron microscopy (Boyde and Stewart 1963), polarization microscopy (Carlström, 1964) and transmission electron microscopy (Nylen 1964). In some countries such as Japan, the close collaboration between dental schools and the JEOL and Hitachi electron microscope companies allowed many dental schools to own an electron microscope and with it employ an expert in enamel ultrastructure on their faculty, resulting in a separate cosmos of enamel research in Japan and unappreciated by the remainder of the scientific world due to language differences.

While there had been several earlier studies related to enamel protein amino acid composition (Block et al., 1949; Stack 1954), more sophisticated analysis techniques during the early 1960ies resulted in detailed and comparable amino acid composition data for enamel from human fetuses (Eastoe 1960, 1963), fetal pigs (Piez 1961), and fetal ox (Glimcher et al., 1961). These analyses identified enamel proteins as proteins with a high content in proline, glutamic acid, histidine, tyrosine, and methionine, while cysteine, hydroxylysine, and hydroxyproline were either absent or only present in very small amounts (Eastoe 1963). Specifically, the low content of cysteine distinguished enamel proteins from keratins, and the low content in hydroxyproline and hydroxylysine together with a high content in prolines suggested that enamel proteins were unique and different from keratins and collagens (Eastoe 1963). These studies used the pliable and soluble enamel matrix as a tool to study the biochemistry of the developing enamel protein phase.

**FIGURE 1:**
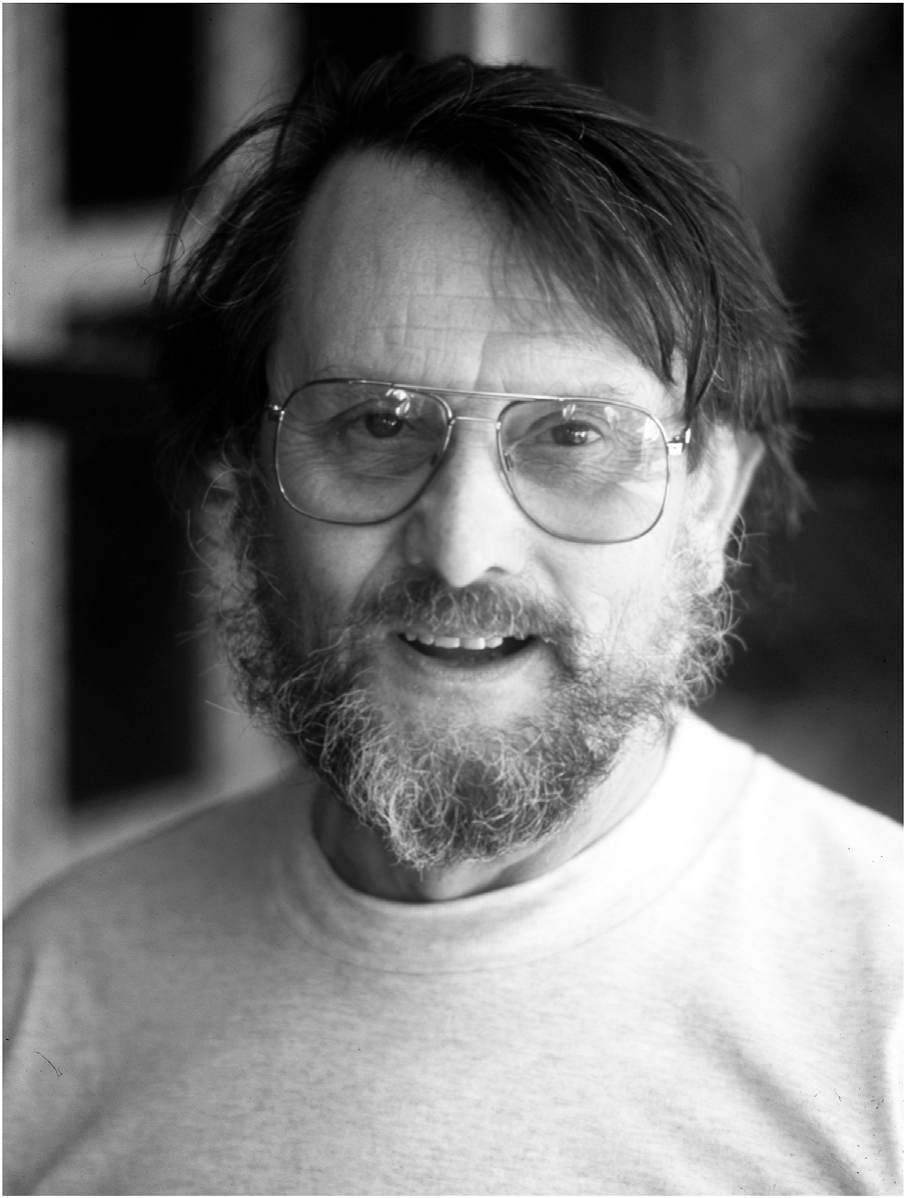
Alan Fincham (1931–2017) at the Center for Craniofacial Molecular Biology in Los Angeles. Photograph taken by the author in 1997.

These classic studies set the stage for further biochemical studies of the developing enamel matrix and the subsequent exploration of its function. In 1979, John Eastoe coined the term “amelogenin” for the major protein in the developing enamel matrix (Eastoe 1979). However, while the physical prevalence of amelogenins in the enamel matrix was suggestive of a functional role, the essential role of amelogenins for enamel crystal formation was only confirmed after a deletion in the amelogenin gene was linked to amelogenesis imperfecta, a congenital enamel defect (Lagerström et al., 1991). Loss of amelogenin also resulted in the loss of enamel in a mouse model (Gibson et al., 2001), and antisense inhibition of amelogenins not only affected enamel crystal growth but also changed enamel matrix organization, suggesting that amelogenin function and enamel crystal growth were intimately linked (Diekwisch et al., 1993). Together, these studies provided the backdrop for the scientific work and intellectual pursuit for one of the most iconic enamel scientists, Alan G. Fincham (Figure 1).

### Enamel research, the British wool industry, and the colonies

Alan Fincham was born in East Preston, West Sussex, the son of a Vicar, on the south coast of the UK in 1931. He was an avid outdoorsman, an active nature lover from an early age. After the war he was involved in the Friends Ambulance National Service, participating in flood rescue relief in Holland and earthquake relief in Crete. After this he spent 2 years at Brighton College of Art, before going on to study at the University of Leeds, graduating with a B.S in Biochemistry and Bacteriology (1959), a M. Sc. in Textile Chemistry 1961) and a Ph.D. in Biophysics (1966). Alan occasionally shared his recollections about the intimate relationship between the University of Leeds and the British wool industry, as it resulted in the sponsorship of several faculty positions that promoted mineralized tissue research in Leeds and the greater Yorkshire region. The wool industry hoped that the new recruits would work on the mineral content of wool, an important parameter for its mechanical properties. Instead, those scientists took a liking to enamel research, among them John Weatherall, Colin Robinson, Alan Fincham and several others. After graduating with a Ph.D., Alan accepted a position as a scientific research officer in the Mineral Metabolism Research Unit at the Medical Research Council in Leeds 1966) and then became a lecturer in the Department of Biochemistry at the Royal Dental Hospital School of the University of London (1967–1969).

After 2 years in London, Alan followed the path of many junior British Scientists of his days and accepted a position through the Commonwealth office as a Lecturer in Biochemistry. In Alan’s case, this position was at the Mona campus of the University of the West Indies, Kingston, Jamaica. Alan had previously visited Jamaica twice before on caving expeditions and as such was familiar with the country and accepted a 3-year contract. Alan moved to Jamaica with his wife Celia and his three children in 1969. He ended up spending 18 years at UWI, first as a Lecturer (1969–1973), then as a Senior Lecturer (1973–1981), and eventually as a Reader (1981–1986). At that time, the University of the West Indies in Mona supported a sprawling Medical School enterprise, and faculty were expected to engage in teaching and research. Alan’s talent as an educator soon became recognized and he was asked to oversee the undergraduate medical curriculum, where he instituted fundamental changes focusing on the student’s learning experiences rather than lectures taught by faculty. Building on his belief in independent learning and his interest in technology he was instrumental in establishing an audiovisual learning library for medical students where students could access recorded lecture lessons, the first of its kind in the West Indies. Continuing his research focus on enamel biochemistry, Alan managed to purchase and operate a high-pressure liquid chromatography apparatus in Kingston, reportedly the only one in the West Indies.

The intricate surface geology of Jamaica became the focus of a third area of Alan’s scholarship. Having explored caves in his native England in Mendip (Wessex) and Yorkshire where he had become involved with cave exploration and rescue—the result of his own rescue after being trapped for 2 hours in a pit he had been exploring with a fellow student while at Leeds. His passion for cave exploration grew throughout his life and he became a leading member and President of the Jamaica Caving Club and spent countless hours tracking and mapping the caves of the island eventually publishing the first comprehensive guide to Jamaican caves “Jamaica Underground” (Fincham, 1977). Even during his Los Angeles years, Alan occasionally traveled back to Jamaica and lead caving expeditions. Ever the outdoors man, he developed an interest in fishing while in Jamaica and took up shark fishing harvesting the teeth of his catch for use in his research. As such it wasn’t uncommon for him to return home on a Sunday evening with the carcass of a Black Tip or Tiger shark strapped to the roof of his old beat-up Volvo station wagon.

### Amelogenin peptide biochemistry at INSERM and at NIDR

With the increasingly violent political situation in Jamaica and three now college-aged children, Alan was looking for opportunities outside of the West Indies. For 2 years (1979–1981), he moved to the National Institute of Dental Research (NIDR; now National Institute for Dental and Craniofacial Research, NIDCR) in Bethesda Washington DC as a Visiting Scientist with John Termine’s group and in 1984 spent a year at the INSERM in Strasbourg, France, with Alain Belcourt. Both stays provided Alan with new scientific opportunities and the interactions necessary for his next move. During this time, Alan published a seminal paper on two functionally significant short amelogenin polypeptides, the tyrosine rich amelogenin peptide (TRAP) and the leucine rich amelogenin peptide (LRAP) (Fincham et al., 1981). At that time, Alan was also successful in isolating individual amelogenin peptides and resolving portions of the complete amelogenin amino acid sequence by N-terminal peptide sequencing (Fincham 1979; Fincham et al., 1983b). Another accomplishment was the amino acid composition determination of human enamel (Fincham et al., 1983a).

Alan’s stay at NIDCR also allowed him to interact with two enamel scientists from the University of Southern California (USC), Drs. Maggie Zeichner-David and Harold C. Slavkin. These interactions eventually resulted in Alan’s recruitment as a Research Associate Professor to the University of Southern California in 1985. The time at USC gave Alan exposure to many likeminded individuals interested in craniofacial and enamel research. Due to its unique faculty retention policies, USC allowed Alan’s three children to study tuition-free on campus, a substantial financial incentive for Alan’s family. As a result, Alan stayed at USC for 16 years, until his sudden departure in 2001.

### The Center for Craniofacial Molecular Biology at USC: Hotbed for scientific discovery

USC provided Alan with multiple opportunities for intellectual and professional growth. When Alan joined, the USC’s Center for Craniofacial Molecular Biology (CCMB) was still located in the Andrus Gerontology Center on USC’s main campus in South Central Los Angeles. Just a few years later, CCMB moved to entirely new facilities as part of USC’s new medical complex in East LA’s Boyle Heights on Alcazar/Soto Street. These facilities represented the state of the art of laboratory architecture at its time, with open lab benches and faculty offices embedded into the lab environment. The Center was the scientific home to approximately 50 lab members, the majority of them postdoctoral fellows from all over the world. There were about a dozen faculty members, such as CCMB director Harold C. Slavkin and several senior scientists, including Alan Fincham, Maggie Zeichner-David, Charles Shuler, Malcolm Snead, and David Warburton from USC Childrens Hospital. Junior faculty members included Ed Lau, Mary MacDougall, Carol Wuenschell and others. At that time, the majority of scientists worked on questions related to tooth enamel, with others focusing on craniofacial biology and lung development. This environment was enriched by weekly lab meetings and presentations by invited speakers from all over the world. These scientific dynamics were an ideal environment for Alan’s scientific and intellectual growth, and he was well respected by students and others alike.

Much of Alan’s initial work was based on enamel protein gel electrophoresis and high-pressure liquid chromatography (HPLC). Alan started very early in the morning, usually on site at 7 am, loaded his samples onto the gel or his HPLC columns and then moved to his office to work on grants or papers. Samples were collected at 2 pm or 3 pm, allowing Alan to reach the freeway prior to afternoon traffic. When working on his HPLC, Alan showed an almost personal relationship with his columns whom he used to address as “guys”. His favorite HPLC column was a unique reversed phase C4 column ideally suited to separate amelogenins from other proteins in the developing enamel. The HPLC apparatus was frequently plagued with service issues, and more often than not, Alan was able to repair the problem without having to rely on the service technician due to his familiarity with the technology. During his Jamaica years, Alan owned the only HPLC apparatus in the West Indies and there were no service technicians available, forcing Alan to address all service issues on his own.

During the early 1990s, USC’s CCMB was a sought-after home for students and postdoctoral fellows seeking to enrich their pre-faculty experience with expert knowledge in craniofacial and enamel biology. Several of these postdoctoral fellows introduced new technologies and tools for amelogenesis research to Alan, who augmented the experience of these junior scientists by providing a wealth of foundational knowledge, intellectual and technical advice. Among these were Jim Simmer, who worked with Alan on the generation of the first recombinant amelogenin, which then became an important tool for enamel studies (Simmer et al., 1994). Another CCMB recruit was Janet Moradian-Oldak, a postdoctoral fellow from Steve Weiner’s lab in Israel, who offered unique insights to the study of biological mineralization.

Janet joined Alan in a quest to resolve the issue of amelogenin phosphorylation, specifically which and how many amelogenin amino acids were phophorylated. At that time, the role of phosphoproteins in enamel mineralization was of great interest as enamel was known to lack collagen, one of the known macromolecules involved in biological mineralization (Curley-Joseph and Veis 1979). Earlier research had identified four “enamel proteins”, among which the 65 and 22 kDa proteins were phosphorylated while the 58 and 20 kDa proteins were not (Guenther et al., 1977). Others had suggested a total of three phosphoserins among phosphopeptides of the enamel matrix (Glimcher 1979). Using a combined approach including reversed phase HPLC, partial acid hydrolysis and mass spectroscopy Alan and Janet determined that there was only a singular phosphorylated amino acid in the bovine amelogenin, the serine in position 16 (Fincham and Moradian-Oldak 1993). This finding provided the basis for later studies related to the role of amelogenin phosphorylation in enamel crystal formation (Kwak et al., 2009; Le Norcy et al., 2018; Shin et al., 2020).

### The “nanosphere theory” and new educational models

**FIGURE 2:**
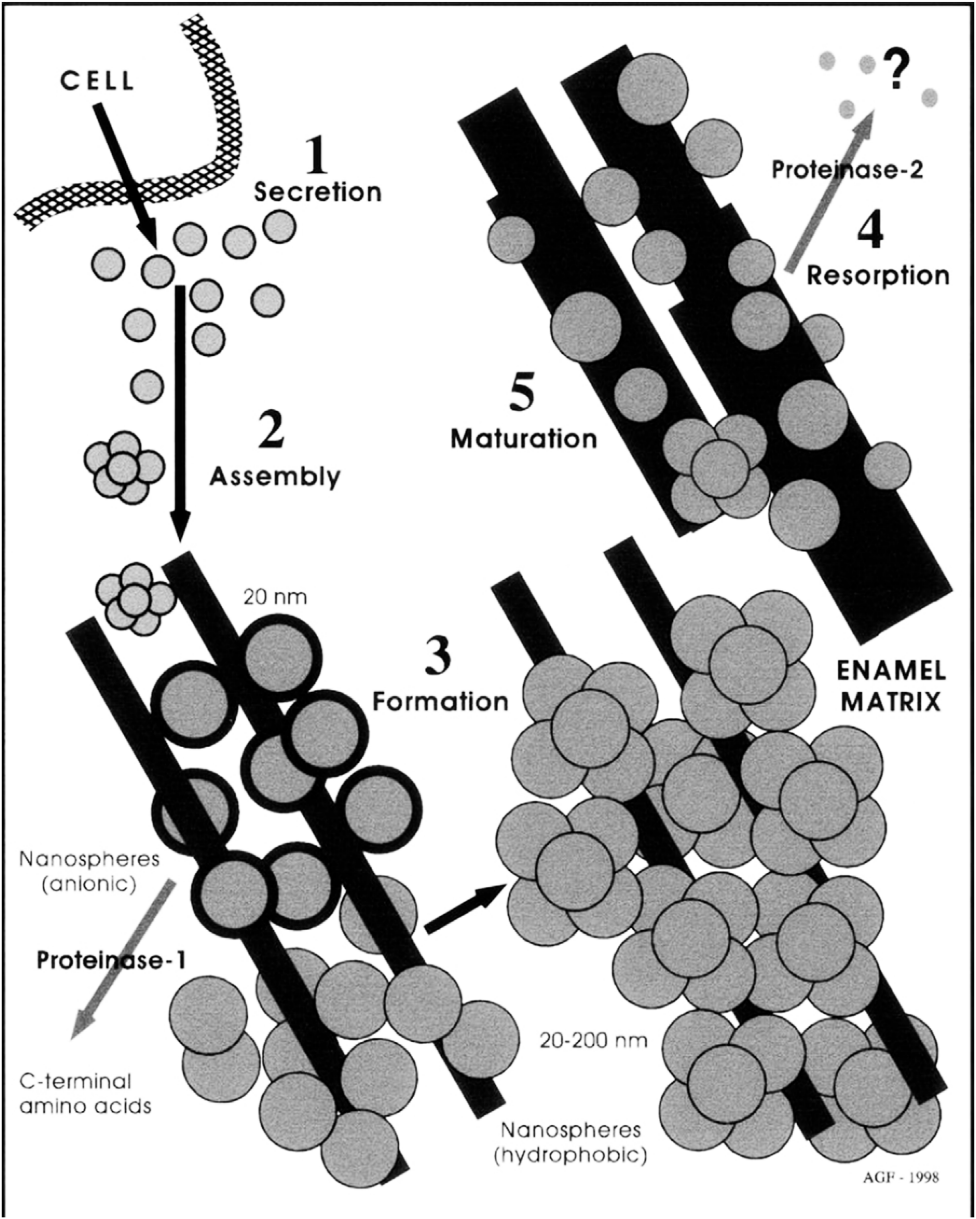
Alan Fincham’s original sketch explaining the “Nanosphere Theory” (from Fincham et al., 1999, Journal of Structural Biology, Elsevier, Figure 8) with permission.

At that time, Alan was examining electron micrographs of the developing enamel matrix (Diekwisch et al., 1993, 1995) and interpreted these micrographs to indicate that the formative enamel matrix was organized into spherical subunits, which Alan called nanospheres (Fincham et al., 1995). Alan was so excited by these spherical structures that he managed to persuade lab director Harold Slavkin to purchase a dynamic light scattering detector and an atomic force microscope (Fincham et al., 1994; Fincham et al., 1995). When all three technologies revealed data consistent with the presence of 20 nm diameter spherical particles, Alan felt sufficiently comfortable to advance the nanosphere theory, a novel theory explaining amelogenesis as a process mediated by the presence of 20 nm diameter spherical amelogenin protein particles (Fincham and Moradian-Oldak 1995). This nanosphere theory greatly influenced our understanding of amelogenesis over the next 2 decades (Figure 2). An excellent history about the emergence of the nanosphere theory has been published in the Journal of Dental Research (Moradian-Oldak 2007).

**FIGURE 3:**
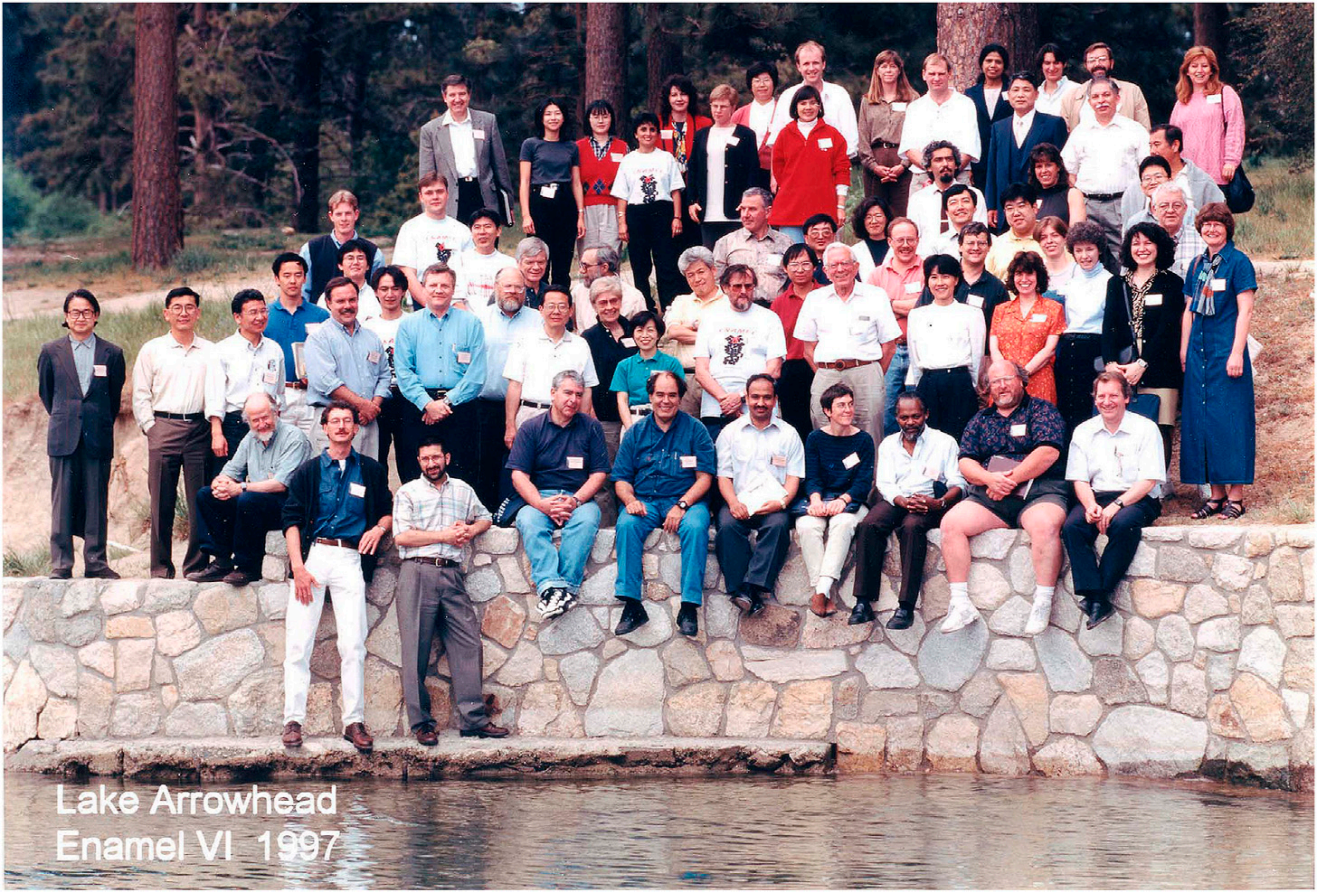
Enamel VI, group picture. Alan is in the second row from the bottom wearing an “Enamel” T-shirt. Photograph taken by the author in 1997.

Alan’s long-standing work in the enamel research field, the prominent progress of his students and his recent scientific leadership established him as the ideal candidate to host one of those legendary enamel meetings. When one of the early doyens of enamel research, Ron Fearnhead moved to Japan, two successive enamel meetings were held in the Far East, Enamel IV in Odawara 1984) and Enamel V in Tsurumi (1989). Following these two very successful meetings there was a general consensus among leaders in the enamel field to convene the next enamel meeting outside of Japan, and it was upon Alan to hold the first enamel meeting on Western soil after almost 20 years. As a conference location, CCMB director Harold Slavkin suggested the UCLA conference center at Lake Arrowhead, 90 miles Northeast of Los Angeles in the San Bernardino Mountains. The conference was held from May 11–15, 1997 and included more than 100 participants from all over the world (Figure 3). Enamel VI became a highlight in Alan’s career as it benefited from Alan’s experience with new educational enterprises and his insight into the current state of enamel research. To facilitate maximum time for scientific exchange, Alan decided to limit presentations to 3 minutes per presenter and five slides maximum. Every presenter was also encouraged to prepare a scientific poster along with the oral presentation. All presentations were followed by approximately ten to 15 minutes discussion, and there were lively discussions every evening in front of the posterboards that lasted late into the night. Enamel research has rarely seen better times.

The faculty at USC’s Center for Craniofacial Molecular Biology (CCMB) were a group of extraordinarily creative individuals who were acutely aware of the limitations of traditional top-down dental education. Partially based on Alan’s experience as head of the undergraduate medical curriculum in Jamaica, CCMB’s faculty decided to embark on a novel approach to dental education focused on student centered learning around clinical problems. In response to their initiative, USC’s Dean Howard Landesman decided to entrust them with a pilot program under the leadership of Alan and Harold’s successor as CCMB director, Chuck Shuler. Creating a new educational program from scratch involved dealing with staffing issues, instructing faculty, designing exam questions, assessing students and guiding students in constructive ways to become competent future dentists. As only the third problem-based dental educational program in North America, PBL initially faced ample criticism and skepticism, especially among USC’s conservative clinical faculty. To address their concerns, PBL student performance and educational outcomes were frequently assessed, and the results of these studies were published in the Journal of Dental Education (Fincham et al., 1997; Shuler and Fincham 1998; Fincham and Shuler 2001). Overall, these studies demonstrated that students in the PBL program performed as well or better than students in USC’s world-renowned traditional program (Shuler and Fincham 1998). Beyond those numerical outcomes, students appeared readily prepared to comprehend complex clinical problems and constructively address issues that were often not accessible to traditional students. There was a real sense of occasion in the early days of CCMB PBL, and students and faculty were proudly aware that they were writing the next chapter in dental education (Fincham and Shuler 2001). Today, the majority of U.S. dental schools have adopted aspects of problem-based or integrated learning to address the need of clinical competencies and the challenge of revised National Board Examinations.

### The final chapter: Sudden departure and new beginnings in Cyprus

By the end of the 1990s, Alan had become one of the foremost enamel scientists and dental educators of his time. Yet, his academic rank had not been advanced beyond that of a research professor. After leaving the West Indies as a Reader, Alan was hired onto USC’s research track, which includes promotions to the rank of Research Professor, but not to full academic ranks such as Associate or Full Professor. After his extensive service as an educational innovator and his dedication toward instituting PBL at CCMB, Alan justifiably expected and requested promotion to the rank of Full Professor. Enamel scientists from all over the world supported Alan’s request and wrote letters of endorsement. Even USC’s conservative faculty were in support of Alan’s promotion. However, once Alan’s promotion was to be implemented Alan received word that his case had been denied. Alan stood in disbelief and complete disapproval of what he considered profound misjudgment. In utter disappointment he packed his belongings into a couple of suitcases and took off to the island of Cyprus in the Mediterranean one week later. His official day of departure from USC was 15 August 2001.

**FIGURE 4:**
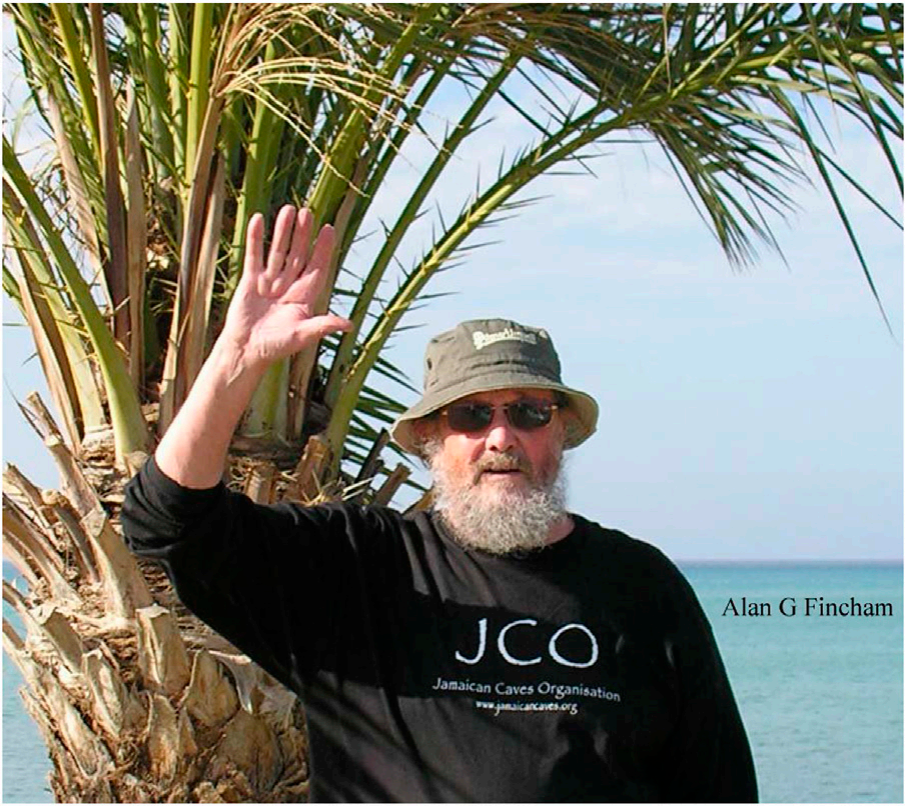
Alan Fincham († 16 May 2017, Cyprus) wearing a T-shirt from the Jamaican Caves Organization. Photograph dated approximately 2015, sent electronically by Dr. Fincham to the author.

In his retirement, Alan developed a new interest in the wildflowers of Cyprus and began the creation of an online database. He also re-engaged with his childhood passion of stamp collecting amassing a collection of thousands of stamps with a primary focus on the stamps of Jamaica and the Caribbean. During the later years of his retirement his arthritic knees damaged through years of walking and hiking in the Yorkshire Dales advanced, making travel and even daily activities increasingly difficult. He no longer was in the spirit of participating in enamel conferences as he loathed to attend as a “has been”. Yet, his interest in enamel research was unbroken: “Please send a copy of the Proceedings when published” he requested. Enamel IX was held in November 2016 and Alan passed away on 16 May 2017, at age 83. A separate copy of the proceedings was never published. Yet Alan’s spirit lives on in the minds of his students and in the greater field of enamel research (Figure 4). Many remember Alan’s enthusiastic mentorship and his encyclopedic knowledge of the enamel field. Most notably, the nanosphere theory of enamel biomineralization will forever be associated with Alan’s name and spirit.

## Discussion

Enamel research has experienced an enormous scientific and technological growth over the past 70 years. Following earlier polarization and electron microscopy studies, emerging biochemical and molecular techniques have allowed for unprecedented insights into how enamel crystals and prisms grow. These insights have been advanced through the pioneering efforts of a number of highly reputed scientists, including the British American biochemist Alan G. Fincham. In the present review we have revisited the scientific context in which Alan performed his protein sequencing studies, identifying LRAP and TRAP polypeptides as major short polypeptides of the developing enamel matrix. We have then described the environment at the University of Southern California that helped Alan promote his nanosphere theory based on converging results from transmission electron micrographs, atomic force microscopy images and dynamic light scattering data. In addition to enamel biochemistry, Alan’s other accomplishments include the establishment of a successful problem-based learning curriculum at the University of Southern California and the publication of the first book on Jamaican caves. These broad-ranging interests and their successful implementation portrait Alan Fincham as a true polymath and as a leading figure who made exceptional and seminal contributions to enamel research. The contributions and Alan’s legacy as a scientist are remembered in the current volume as we once again publish papers related to an enamel meeting.
